# Speech recognition software and electronic psychiatric progress notes: physicians' ratings and preferences

**DOI:** 10.1186/1472-6947-10-44

**Published:** 2010-08-25

**Authors:** Yaron D Derman, Tamara Arenovich, John Strauss

**Affiliations:** 1 Information Management Group, Centre for Addiction and Mental Health, 1001 Queen St. West, Toronto, Ontario, M6J 1H4, Canada; 2 Biostatistical Consulting Service, Centre for Addiction and Mental Health, 33 Russell St., Toronto, Ontario, M5S 3M1, Canada

## Abstract

**Background:**

The context of the current study was mandatory adoption of electronic clinical documentation within a large mental health care organization. Psychiatric electronic documentation has unique needs by the nature of dense narrative content. Our goal was to determine if speech recognition (SR) would ease the creation of electronic progress note (ePN) documents by physicians at our institution.

**Methods:**

Subjects: Twelve physicians had access to SR software on their computers for a period of four weeks to create ePN. Measurements: We examined SR software in relation to its perceived usability, data entry time savings, impact on the quality of care and quality of documentation, and the impact on clinical and administrative workflow, as compared to existing methods for data entry. Data analysis: A series of Wilcoxon signed rank tests were used to compare pre- and post-SR measures. A qualitative study design was used.

**Results:**

Six of twelve participants completing the study favoured the use of SR (five with SR alone plus one with SR via hand-held digital recorder) for creating electronic progress notes over their existing mode of data entry. There was no clear perceived benefit from SR in terms of data entry time savings, quality of care, quality of documentation, or impact on clinical and administrative workflow.

**Conclusions:**

Although our findings are mixed, SR may be a technology with some promise for mental health documentation. Future investigations of this nature should use more participants, a broader range of document types, and compare front- and back-end SR methods.

## Background

Speech recognition (SR) has taken decades to mature to the point where it can be used in medicine. Acceptance of the SR has slowly increased as hardware and software have matured so that the technology 'adjusts' to the user rather than vice versa [1]. The introduction of continuous speech systems - which allow the user to speak in his/her normal vernacular and rate of speech - has increased the potential that this technology can enhance the efficiency and quality of creating documentation without negatively impacting the user's time. Bergeron [2] has provided an overview of the various options for implementing voice recognition by individual clinicians.

A majority of the earliest reported uses of SR in medicine were in radiology [3-5]. Early continuous speech recognition resulted in semantic accuracy up to 81% [6], but still was not good enough for clinical use. By 1997 SR and medical knowledge bases were used to produce structured reports [7]. Another study comparing three different SR applications against each other concluded that increasing computer power, affordability, and software sophistication was making the replacement of transcription with SR more feasible [8].

Issenman and Jaffer [9] have proposed that most successful implementations have been performed in radiology or emergency services because these specialties lend themselves to SR because they use many highly repetitive phrases. Radiology departments have reported reduced turnaround times and decreased costs for both front-end (edited by the physician) and back-end (edited by an editor/transcriptionist) solutions [10-12]. Similar benefits have been reported in emergency services [13] and pathology [14]. Mohr et al [15] conducted a randomized comparison of Endocrinology and Psychiatry outpatient notes transcribed by transcriptionists and found that SR reduced the transcriptionists' productivity. In a case study analysis in a large pediatric hospital, staff perceived that back-end SR improved turnaround times and accessibility of the reports, and reduced costs without negatively impacting workflow [1].

Along with cost and turnaround times, studies have measured clinician efficiency, clinician satisfaction and dictation accuracy. Borowitz [16] found that the total time taken to dictate and edit a pediatric gastroenterology outpatient clinical note using SR took 15% longer than a transcriptionist typing notes recorded on a hand-held recorder. However, all SR generated notes were completed within 48 hours of the patient visit, whereas only 24% of transcribed notes were completed in this time frame. Another pediatric gastroenterologist reported that dictating and self-editing a clinical note took 200% longer than transcription by a skilled transcriptionist and cost 100% more [9]. In a Danish hospital that had replaced transcription with SR in all clinical specialties to reduce documentation turnaround times, physicians surveyed felt that they spent more time creating medical records and clinical documentation quality had declined [17]. Pezzullo et al. [18] found that using SR to create radiology reports in one non-academic setting increased radiologists' feelings of frustration and introduced more errors into reports and increased costs as compared to conventional transcription services. Another study noted that more than 20% of SR-created reports contained potentially confusing errors, and most radiologists believed that error rates in reports were much lower than they actually were [19]. In summary, SR technology has provided mixed results in various clinical settings.

The purpose of this study was to determine if SR would ease the creation of electronic documents by physicians in support of mandatory use of electronic progress notes (ePN) within a large mental health care organization. The literature reviewed did not address mental health clinical documentation generated with SR and edited by physicians. We studied physicians' perceived value and willingness to adopt SR in relation to the usability, data entry time savings, impact on the quality of care and quality of documentation, and the impact on clinical and administrative workflow, as compared to the existing methods for data entry.

The Centre for Addiction and Mental Health (CAMH) is Canada's largest academic mental health hospital. Nine clinical programs offer inpatient and/or outpatient services from three main sites; there are 35 satellite locations throughout the province. CAMH has 565 inpatient psychiatric beds, 1400 clinical staff, over 130 full-time medical staff, and the largest national postgraduate psychiatry residency program. In fiscal year 2007/08, CAMH provided services for 3,698 inpatient visits and 436,193 outpatient visits.

The clinical applications infrastructure at CAMH includes hospital-wide electronic progress, group and care plan notes, a document management system, a dictation/transcription system for reports, electronic laboratory orders and results, and a small barcode medication administration pilot on two inpatient units. There is no computerized medication order entry or radiology functionality.

By April 2009, all CAMH clinicians were required to document ePN using a web-based application for clinical documentation. CAMH medical staff responded affirmatively to a 2007 survey that they would like to evaluate various data entry technologies to assess if these technologies could assist in efficient production of electronic documentation. SR was selected for evaluation in the first phase of the CAMH physician electronic documentation (e-Doc) project. The CAMH Research Ethics Board approved the research protocol and participants consented to participate.

Requirements for clinical documentation by physicians in Ontario are dictated by the College of Physicians and Surgeons of Ontario (CPSO). The Ontario government's Medical Review Committee (MRC) audits claims submitted by physicians, and consists of physicians nominated by the CPSO and six members of the public. For progress notes in Ontario, a SOAP (Subjective/Objective/Assessment/Plan) format is recommended. For groups, a separate group note is required for each patient-client participating in a group.

At the time of the project, ePNs were being implemented hospital-wide in a staged approach. Because of this, at the time of the study, some participants were already typing ePNs, while others still wrote progress notes on paper, or dictated them for transcription. The SR software (SRS) was compatible with ePN, so notes could be dictated directly into the application instead of typed. Those not yet using ePN were supplied with a Microsoft Word (Redmond, WA) template to dictate into. These progress notes were then printed out and included in the paper-based clinical record. Participants were permitted to use the SR for other applications as well.

## Methods

Data was collected, over a 6 month period from July 2008 to January 2009, including pre- and post-usage surveys and group debriefing sessions. SRS was installed in staggered fashion in September/October 2008; deinstallation was between November/December 2008, also staggered.

**Figure 1:**
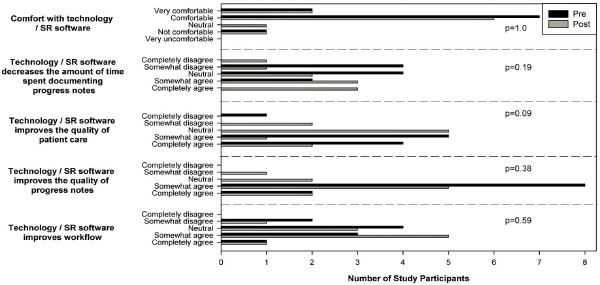
**Physicians' ratings pre- and post-speech recognition**. Physicians' ratings of pre- and post-speech recognition (SR) comfort with technology/SR, time efficiency, quality of care, quality of notes and improved workflow. Pre-SR bars are black. Post-SR bars are lighter in colour. The x-axis is the number of participants with a given rating. e.g. One participant reported a post-usage rating of "Neutral" re Comfort with technology/SR.

Twelve physicians (9 male, 3 female) of the CAMH medical staff were selected to participate in the project to represent the broader CAMH medical community. All were psychiatrists, except for one primary care physician. They were selected from four clinical programs, of which two had been using ePN for at least 6 months. Selection was based on expressed interest, comfort with technology and perceived need. All participants had outpatient clinical duties, and five also had inpatient duties. Nine reported 'comfortable' or 'very comfortable' as their level of comfort with technology in the pre-usage survey (described below) (Figure 1).

Each participant identified the computer s/he used most often to create progress notes. Where necessary, this computer was upgraded to meet the minimum requirements for the SRS listed on the product vendor's website. A leading SRS product was installed by an established SR vendor - the vendor provided 30-60 minutes of individual training to each participant and pager and email contacts for technical support. SRS audio files were stored on the clinician's network drive folder and not shared. The focus was on using SRS to assist with the implementation of ePNs, which are the bulk of clinical text entry for CAMH clinicians.

The installations were staggered to accommodate participant and vendor availability. Participants were encouraged to use the software as often as possible to provide a thorough evaluation on behalf of their colleagues. Several participants experienced technical issues using the software, which impaired their ability to use it throughout the installation period - the vendor resolved all issues reported. All participants were given at minimum four weeks to trial the software.

Pre- and post-usage surveys were administered to compare baseline opinions about technology with post-usage opinions about SR. The pre-usage evaluation began in July 2008. Ten of the twelve participants completed a ten-question pre-usage survey that was designed for the evaluation. After the SRS had been removed from all the participants' computers, all twelve participants completed the post-usage survey.

Additionally, post-usage group sessions were held to collect additional feedback and opinions about the potential value of SR to the broader CAMH medical community.

Nine participants were available for the post-usage debriefing sessions to elaborate upon their survey responses. Two participants that could not attend a debriefing session provided answers to the debriefing questions via email.

We hypothesized that physicians would benefit from using SR and that this would be reflected by an increase in measures of perceived value of SR over the course of the study. Changes in scores among the ten participants that completed both surveys were assessed through a series of Wilcoxon signed rank tests. Analysis was conducted using SAS (Cary, NC) version 9.1.3.

## Results

Our results are summarized in Table 1 and Figure 1. **Table **1 contains post-usage frequency of SR use, computer availability and preferred data entry method.

**Table 1:** Frequency of SR use, computer availability, and preferred data entry method

Question	Response	n
Frequency of SR use (% of all progress notes created)	0-20%	3
	20-40%	3
	40-60%	2
	60-80%	2
	80-100%	2
		
How accessible are computers when you need to make a progress note?	Rarely	2
	Sometimes	1
	Mostly	2
	Always	7
		
Which method of data entry do you prefer?	Handwriting	3
	Keyboard	3
	SR	5
	Digital dictation	1

**Frequency of SR use **data showed three participants used SR for 20% or less of their progress notes; two participants used SR for 80% or more of their progress notes. The rest were distributed in between. Concerning **computer accessibility**, nine physicians indicated computers were mostly or always accessible and three stated computers were sometimes or rarely accessible when they were needed to create a progress note. Our findings on **preferred method of data entry **demonstrated fully half (6/12) of the participants preferred SR. Five participants preferred to use SR for progress notes, one preferred SR assisted by dictation into a hand-held digital recorder, three preferred keyboard typing, and three preferred handwriting.

We found no statistically significant difference pre- or post-usage for comfort with SR software, data entry time savings, quality of care, quality of documentation, or impact on clinical and administrative workflow. More detail is provided below. (See Figure 1.)

### Comfort with technology and SR

As a group, 9/10 post-usage survey completers rated themselves nearly identical to pre-usage ratings (Figure 1). There were very few requests made for support service throughout the project. At the debriefing sessions, all participants agreed that the software was easy to use and they experienced next to no technical issues - one physician reported difficulty with SR where the cause was a desktop computer with hardware problems.

Some participants found that they did not achieve a satisfactory recognition of their speech by the software. Potential causes that were observed included: understanding of how to train the software, speech accent, environmental noise, or minimal opportunity to use it during the pilot.

One participant discovered that, although he considered himself a fast typist, using SR "*feels like less effort than typing*". A different participant felt it preferable to take time to think before typing and create fewer edits than to have to scrutinize the SR documents for mistakes. "*It is easier to take my time and type what I want to type than to correct*." One of the participants who indicated in the post-usage survey that SR was not the preferred data entry method, commented *"I did not realize all this while I had the SR but now that I do not have access to it I am missing it."*

### Data entry time savings

Overall, participants did not find that SR decreased the amount of time spent documenting progress notes as compared to their current method of data entry or other types of technology (p = 0.19, Figure 1). Still, one participant found that his overall client throughput increased 20% as a result of using SR; the clarity of his notes improved because he used fewer acronyms. Those that considered themselves good typists felt that typing was more efficient for short clinical documents such as progress notes. Also, some had (independent of SR) begun to type during the client interview, reducing the time required to create a progress note after the interview.

### Quality of patient care

Participants tended to rate overall technology as having more of a positive impact on quality of patient care than SR, but it was not statistically significant. (p = 0.086). Participants did not use SR while providing patient care. However, one participant who conducts a high volume of outpatient visits noted that *"I could spend more time talking to clients, as I spent less time typing notes"*.

### Quality of clinical documentation

Over the course of the study, there was no difference in perceived quality of documentation with SR compared to overall technology (p = 0.375). In the debriefing sessions, participants noted that there are errors introduced into the documents by SR that are more subtle, therefore "*you need to proofread quite carefully*". Increased vigilance in proofreading was considered a negative outcome of SR, and not a positive contributor to the overall quality of the clinical documentation. In contrast, one participant explicitly observed improved progress note quality.

### Clinical workflow

In the pre- and post-usage surveys, participants reported that SR did not significantly improve their clinical and administrative workflow over current data entry methods or other types of technology (p = 0.59). In the pre-usage survey, most participants reported that a computer is always accessible when they need to create progress notes, but in the post-usage survey, several participants found that they were delayed in creating progress notes because the computer with the SR software was not accessible (Table 1).

Many participants stated that they began writing paper-based progress notes during a patient interview and then completed them afterwards. With SR, they needed to create the entire note after interview because they would not dictate in front of patient. Finally, participants with heavy inpatient work found that SR was not conducive to inpatient workflow. This was due to the noisy environment and limited access to computers during and after a client interview. Several participants expressed interest in using a dictation device that captures a voice recording file for upload to SR software to convert the voice file to text; such devices are available in the marketplace.

## Discussion

To summarize, while half of the twelve participants favoured the use of SR for electronic progress notes over other data entry methods, there was no clear perceived benefit to SR regarding time savings, quality of care, quality of documentation, or impact on clinical and administrative workflow. The results of the project suggest that some physicians are more likely to find SR useful in creating mental health progress notes than others. The literature reviewed also reported mixed results for the benefits of SR technology in clinical workflow of other medical specialties [1,3,4,9-19].

The following themes arise from our observations, and from published reports. Four factors may contribute to physician use of SR for mental health progress notes:

**• Attitude towards one's ability to learn to type and one's typing efficiency **- In the e-Doc project, those that were accustomed to typing - whether they were good typists or not - were less likely to prefer SR. Good typists found that for short progress notes, typing was quicker and more conducive to inpatient workflow. Slow typists that were accustomed to ePN progress notes also did not necessarily prefer SR. Physicians that have not 'accepted' typing will be more likely to adopt SR.

**• Perceived value proposition **- Quantifiable administrative benefits of SR were assessed in previous studies - most reported SR successes were 'back-end' implementations where the physicians did not have to 'invest' time in order to receive these administrative benefits. We compared the perceived 'investment' of using 'front-end' SR to create and then self-edit the clinical documents to typing electronic progress notes. Participants who judged that SR adequately provided value in return for the effort involved in using it were most likely to prefer it.

**• Learning curve tolerance **- Issenman and Jaffer [9] reported that only one of four initial study participants overcame frustrations in training the software to arrive at a level of mastery necessary to conduct the trial. In the e-Doc project, we found three of six participants that did not prefer SR reported technical and/or training issues with the software. One participant invested seven hours reviewing and training the software, and afterwards found SR worked better. Some individuals are simply more tolerant of learning new technologies than others. Bergeron proposed "I suggest you pick up a $100 general-purpose voice-recognition package from your local microcomputer center and work with it for a week. I've found that it's a love-it or hate-it affair [2]."

• **Homophily **- Parente, Kock and Sonsini propose that "the extent that physicians are homophilous, or share beliefs, education, social status and other similar attributes, will influence their attitude toward the adoption and implementation of speech-recognition technology" [1]. Alapetite et al. [17] reported that physicians' perception of their colleagues' assessments of SR correlated significantly with their own overall assessment of SR. Most of the participants were from different CAMH clinical areas or services and rarely interacted with one another, making it difficult for us to comment.

Three potentially confounding events occurred during the project. First, three of the original twelve participants dropped out of the project before the software was installed. Three new participants agreed to join the e-Doc after the initial planning phase. Ultimately, twelve participants had SR installed throughout the e-Doc project, as originally planned. Second, due to schedule limitations (e.g. vacation, travel, number of work days at CAMH), SR usage time tended towards being shorter. A longer period of time for physicians to learn the software, optimize their voice profile and learn the voice interface would have been helpful. However, time and resource constraints made for a duration briefer than initially planned. Longer duration of use could have produced more significant positive differences. Finally, three of the participants revealed that they had experimented with SR technology prior to the project. This may have influenced their opinions about the value and usability of the software.

Some limitations to our findings should be acknowledged. Importantly, our participants were not randomly chosen, so they may not be representative of the larger mental health medical staff community. Second, our sample size was very modest, owing to time and resource constraints. Third, we did not collect the ages of the participants. Fourth, we asked participants to evaluate SR for only one type of document. The perceived value of SR may have differed had the study been framed as using it for all types of clinical and administrative documentation. Finally, we installed SRS on one computer per participant. If it were more readily available on several computers or through the use of a digital dictation recorder that integrates with the SR software, the ease of access may have increased use.

Another caveat involves the measures of quality we used. Quality of care and quality of documentation ratings were subjectively rated by participants: limitations include differing personal definitions of quality and variation in expectations of SR. Another limitation of our investigation is the existence of two modes of progress note entry - in the programs that were live with ePN and in the programs that were still using paper progress notes. Many physicians in the ePN group had bee documenting electronically for several months, and would have been more comfortable with electronic documentation. Not surprisingly, SR was preferred more by the ePN group: Of the eight post-usage respondents that had access to ePN, five favoured SR or digital dictation; of the four post-usage respondents who used Word, one favoured SR.

We did not perform formal usability testing prior to our study for two main reasons. Early SRS adopters among the medical staff who reported good usability and productivity gains. Moreover, a neighbouring hospital's psychiatry department had successfully replaced their transcription service with SRS using the same software and vendor as we did. Therefore, we thought SR methods were sufficiently usable, and wanted to test them live.

We did obtain some unanticipated observations with "in situ" clinical use, namely i) good typists found that for short progress notes, typing was quicker and more conducive to inpatient workflow and ii) participants found that their fellow clinicians were detecting some word errors in the SRS-generated notes, likely because SRS can yield correctly spelled words that are contextually incorrect.

While the limitations may impinge on how generalizable our findings are, our results are supported by other investigations [14,17,18].

## Conclusions

We observed that half of physicians that evaluated SR favoured the use of SR for creating mental health progress notes over other data entry methods. Attitude towards typing, anticipated 'return on investment' and learning curve tolerance appear to be good heuristic indicators of adoption likelihood of SR by psychiatrists. Additional research should use larger samples, a broader range of document types, and compare front-end and back-end SR methods to examine how SR might optimally assist with mental health documentation. Collecting additional data variables and mobile dictation will help to determine if physician age, typing skills or mobile SR are associated with preference for or perceived value of SR.

## Competing interests

The authors declare that they have no competing interests.

## Authors' contributions

YD and JS conceptualized and designed the study, and co-wrote the manuscript. YD carried out the data collection. TA completed the data analysis and created the graphics. All authors reviewed and provided feedback on the final version of the manuscript.

## Pre-publication history

The pre-publication history for this paper can be accessed here:

http://www.biomedcentral.com/1472-6947/10/44/prepub
